# Nanostructured Three-Dimensional Percolative Channels for Separation of Oil-in-Water Emulsions

**DOI:** 10.1016/j.isci.2018.08.004

**Published:** 2018-08-13

**Authors:** Jian Jin, Xiaoli Zhao, Yong-Hua Du, Mei Ding, Chengjie Xiang, Ning Yan, Chuankun Jia, Zheng Han, Lidong Sun

**Affiliations:** 1 State Key Laboratory of Mechanical Transmission, School of Materials Science and Engineering, Chongqing University, Chongqing 400044, China; 2 Institute of Chemical and Engineering Sciences, A*STAR, 1 Pesek Road, Jurong Island, Singapore 627833, Singapore; 3 College of Materials Science and Engineering, Changsha University of Science & Technology, Changsha 410114, China; 4 State Key Laboratory of Rolling and Automation, Northeastern University, Shenyang 110819, China; 5 Shenyang National Laboratory for Materials Science, Institute of Metal Research, Chinese Academy of Sciences, Shenyang 110016, China; 6 School of Material Science and Engineering, University of Science and Technology of China, Anhui 230026, China; 7 Key Laboratory of Advanced Energy Materials Chemistry (Ministry of Education), Nankai University, Tianjin 300071, China

**Keywords:** Chemical Engineering, Separation Science, Materials Science, Composite Materials

## Abstract

Separation of oil/water mixtures has been one of the leading green technologies for applications such as oil recovery and water purification. Conventional methods to separate oil from water are based on phase separation via physical settlement or distillation. However, challenges still remain in the effective extraction of micron-sized oil droplets dispersed in water, in which case gravity fails to work as separating force. Here, we conformably decorate porous titanium (average pore size 30 μm) with superhydrophilic nanotubes. The resulting three-dimensional superhydrophilic micro channels thus provide a driving force for oil-water separation at the nanotube/emulsion interface, enhancing significantly the water infiltration rate. The high efficiency (>99.95%, with oil droplets of average diameter 10 μm) and strong mechanical durability make the structure a reusable oil/water separator. Our findings pave the way for future applications of oil-in-water emulsion separation, which can be readily scaled up for massive demulsification.

## Highlights

• TiO_2_ nanotube arrays percolatively cover entire 3D channels of porous Ti foams
• The above-mentioned superhydrophilic foams separate micron-sized oil droplets from water
• Separation mechanism of oil-in-water emulsion is studied by the XANES

## Graphical Abstract

**Figure:**
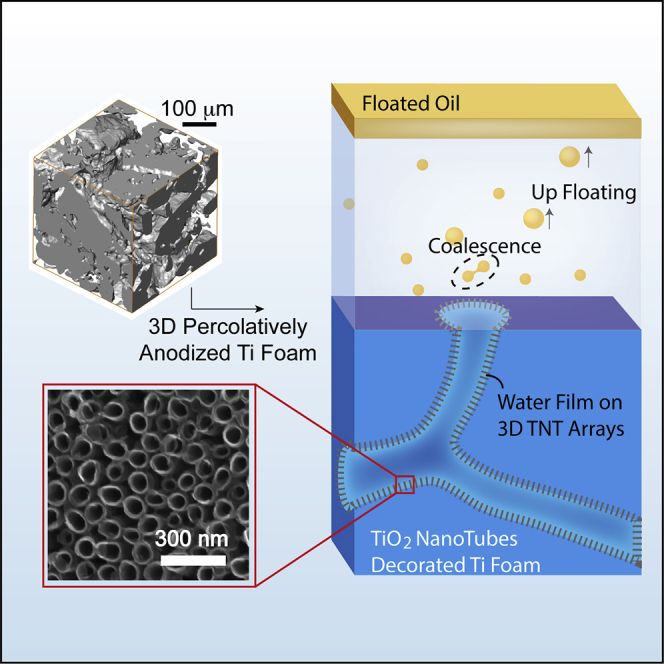

## Introduction

Separation of oil from water has been an issue that has developed along with the development of modern technology (Li et al., 2013, Zhou et al., 2018). For example, petroleum manufacturing process has aroused environmental concerns such as oil waste and water pollution (Vlasopoulos et al., 2006). Recent advancement of green technologies has also boosted the need of recovery of oil from water for domestic uses such as kitchen waste recycling (Tuteja et al., 2007, Shimizu and Tanaka, 2017). It is known that by utilizing gravity (Dong et al., 2014), centrifugal force (Krebs et al., 2012), electrochemical means (Ristenpart et al., 2009), or adsorption (Ge et al., 2017), oil/water mixtures can be separated in a macro scale.

However, those conventional methods often fail to work when it comes down to the micro scale, which is most often the case in practical applications (Zeng et al., 2017). To mitigate this, treatments such as chemical agents are often applied in demulsification technology (Jiang et al., 2017). To achieve high-efficiency separation of micron-sized oil droplets in water (i.e., oil-water emulsion), major challenges remain in the high porosity and superhydrophilic surface as concomitants of the material itself. Recently, emerging designs of oil-water emulsion filters, such as organic membranes (Luo and Liu, 2017) and surface-functionalized metal meshes (Zhou et al., 2017a), have become popular in the field of oil/water separation research. Nano-arrays mimicking cacti surface are also reported to be candidates for the effective collection of micron-sized oil droplets from water (Ju et al., 2012). Nevertheless, they either suffer from poor mechanical durability or from an impotent filtering efficiency. To date, a single reusable demulsificator that combines the merits of mechanical durability, high efficiency, and high throughput has been missing.

Among the reported demulsificators, self-organized anodic TiO_2_ nanotubes (TNTs) vertically grown on non-planar titanium substrates have attracted tremendous interest because of the superb water wettability (Balaur et al., 2005, Xiang et al., 2017). For example, TNT-covered titanium meshes, wires, and tubes have been used not only in oil/water separation (Sun et al., 2014, Liao et al., 2012) but also in fields such as organic matter degradation (Liao et al., 2012), flexible solar cells (Wen et al., 2016), and Li-ion battery systems (Zhang et al., 2014). The growth mechanism of vertical TiO_2_ nanotube arrays (TNTAs) on titanium foams is yet elusive. The reported oil/water separation methods based on TNTAs are mostly limited to the outer surface modification on top of the titanium foams (Li et al., 2015c) and only with low porosity because of the poor oxidation kinetics condition in the micro-pores (Atencia and Beebe, 2005, Wei et al., 2000).

In this work, we show that by developing a three-dimensional (3D) percolative anodization technique, high-porosity titanium framework with thickness reaching a few millimeters can be conformably decorated with superhydrophilic TNT vertical arrays. These 3D percolative superhydrophilic micro channels can serve as “highways” for water but prevent the transport of oil droplets (average size 10 μm, see Supplemental Information). It thus leads to a low-cost reusable oil/water separator with ultrahigh efficiency (>99.95%) and strong mechanical durability.

## Results

### Fabrication of 3D Percolative Superhydrophilic/Superhydrophobic Micro Channels

**Figure 1:**
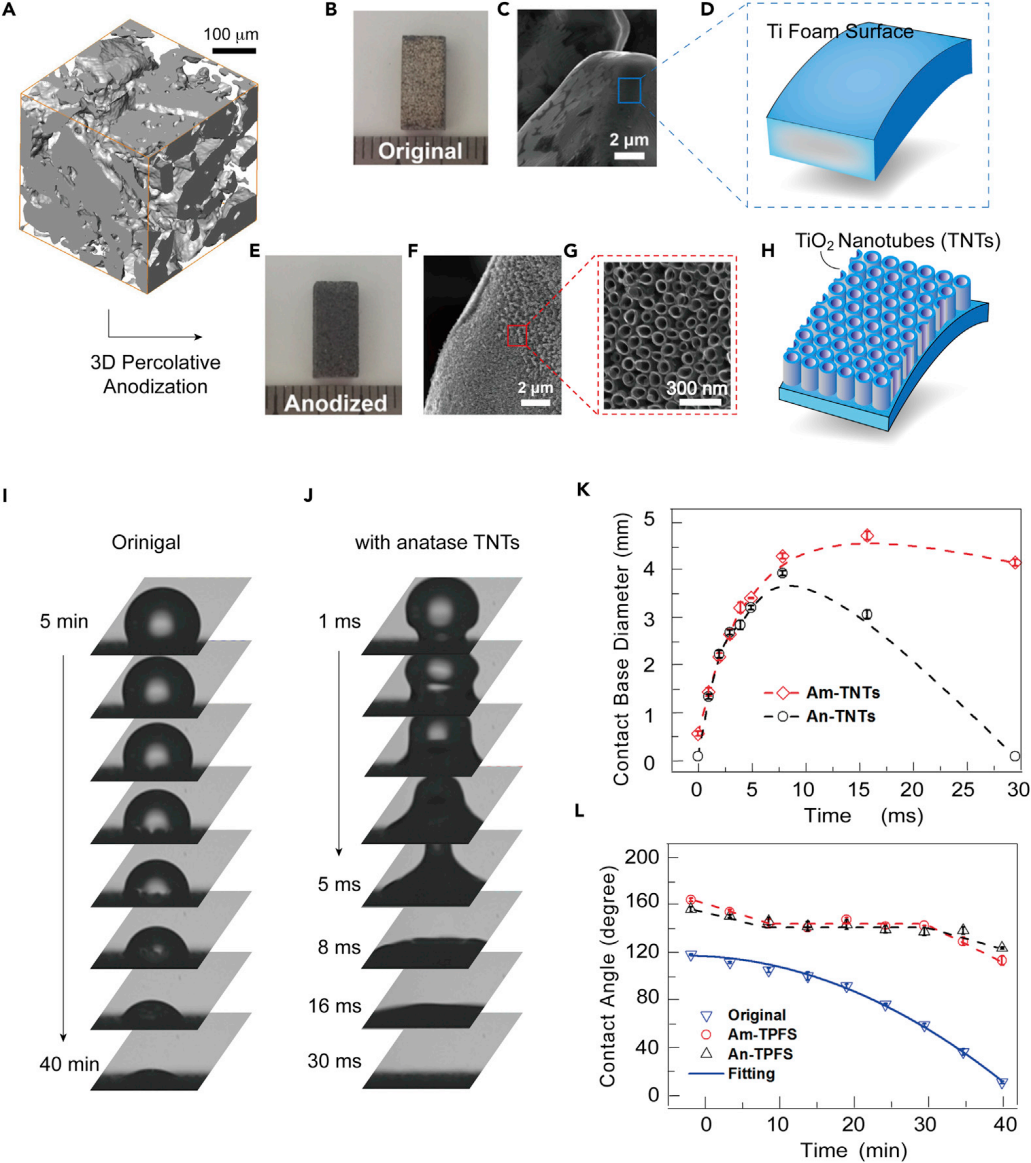
Morphology and Wetting Properties of 3D-TNTAs (A) 3D topological morphology of the pristine Ti foam, obtained by the 3D micro-CT technique. (B–J) Photograph (B, E) and scanning electron micrographs (C, F, and G) of an original Ti foam (B, C) and that conformably coated with TNTs (E–G). Corresponding illustrations are given in (D) and (H), respectively. Time sequence of video frames showing the evolution of water droplet on original (I) and anatase-TNT-coated (J) foams. (K and L) Contact base diameter (K) and contact angle (L) as a function of time for superhydrophilic (amorphous nanotubes, Am-TNTs; anatase nanotubes, An-TNTs) and superhydrophobic (amorphous nanotubes with TPFS treatment, Am-TPFS; anatase nanotubes with TPFS treatment, An-TPFS) foams. Each of the data point in (K) and (L) was based on at least three samples. See also Figures S1−S7, and S10, and Table S1.

Anodization is a commonly used method to produce vertically aligned titania nanotube arrays on Ti surface, which can further tailor the wettability of Ti into a superhydrophilic state (Xiang et al., 2017). Here, to achieve high efficiency, as well as to maintain the mechanical strength of the demulsificator, we chose Ti foam with high porosity and a total thickness of 3–5 mm. As confirmed by 3D micro-computed tomography (CT) in Figure 1A, pore sizes in the foam matrix are of the order of 20–30 μm. The original Ti foams exhibit metallic luster and flat micro-morphology upon a closer look at the surface of the micron-sized pores (Figures 1B–1D).

Open-ended TNTAs are then formed on the titanium foam by anodization in ethylene glycol solution (see Methods). Figure S1C shows the cross-sectional morphologies of the Ti foam with TNTAs grown at 40 V for 40 min in electrolytes with different water contents. Field emission scanning electron microscopic characterization indicates that the nanotubes initiate at the superficial regions (noted as zone A, see Figure S1B) of the Ti foam.

However, rather than nanotubes, titanium oxide films were grown at the inner region (noted as zone B) mostly due to the poor ion diffusion condition in the micro-pores. This outer surface growth of TNTAs on titanium foams was reported previously and has hindered its application in oil/water separation due to poor efficiency (Li et al., 2015c). We have tested different parameters in the anodization process (Supplemental Information), and an optimized anodization duration of 3 hr was used in the electrolyte (n = 4) to synthesize the 3D percolative superhydrophilic TNTAs that conformably cover the high-porosity titanium framework with total thickness reaching a few millimeters. As shown in Figure 1E, after the 3D percolative anodization, the Ti foam with TNTAs presents a dark color (See also Figure S2). The scanning electron micrographic morphologies in Figures 1F and 1G show that the vertically aligned TNTs are open-ended and that the nanotube diameter is distinctly smaller at the top than the bottom, which is assigned to a longer etching duration at the top in the electrolyte and also to the curved channels (see Figure S3).

After a thorough 3D percolative anodization process, we found that both the outer surface (zone A) and inner regions (zone B) can be fully and conformably decorated with vertical TNTAs, as illustrated in Figure 1H. It is noted that, for 3-hr anodization, the average lengths of the nanotubes in zone A are saturated at about 1.8 μm, whereas those in zone B are 0.95 μm, which can linearly increase with longer processing time due to the lagged balance time (see Figure S1E). In addition, the superhydrophilic-to-superhydrophobic transition can be achieved with a chemical modification by *n*-hexane (99%, Adamas) containing 0.5 vol% trichloro (1H,1H,2H,2H-perfluorooctyl) silane (TPFS) on the 3D TNTAs. As will be discussed in the coming sections, for the hydrophilic foam, water infiltration rate reached 173 μL⋅s^−1^, which is 5 orders of magnitudes higher than the original foam.

### Surface Wettability Characteristics of Ti Foams with Different Surface Functionalization

In the following, we mainly focus on 5 different types of Ti foams, i.e., the original (without nanotubes, Type I), the as-prepared 3D percolative anodized amorphous TNTAs-decorated Ti foam (superhydrophilic nanotubes, Type II), the annealed 3D percolative anodized TNTAs-decorated Ti foam (superhydrophilic nanotubes, Type III), the TPFS-treated amorphous TNTAs-decorated Ti foam (superhydrophobic nanotubes, Type IV), and the TPFS-treated annealed TNTAs-decorated Ti foam (superhydrophobic nanotubes, Type V). As shown in Figure S4, drop impact tests were performed to verify the surface wettability characteristics of each type of foam. The visual test process of types II and III is recorded in Video S1. The results indicate that, for the original titanium foam, the water droplet adhered to the foam and presented a viscous hydrophobic state. However, the water droplet instantly spread on the Type II/III foams, due to the composite 3D structure of micro-pores and the superhydrophilic TNTAs. A superhydrophobic behavior was observed on the Type IV/V foams, as the droplet rolls freely on the foam, similar to the behavior of water droplet on a lotus leaf.

Video S1. Drop Impact Tests Performed to Verify Surface Wettability Characteristics of Type II and Type III Foams, Related to Figure 1

The evaluation of sessile water droplet on foams with different wettability is shown in Figures 1I, 1J, and S5. The water droplet infiltrated after about 40 min on the original foam (Type I). For the Type I foam, there was an evaporation effect during the capillary absorption of water droplet. The evaporation rate can be estimated based on the tests on superhydrophobic foams (Types IV and V); under such conditions, evaporation dominates the mass loss with negligible infiltration. The droplet volume was decreased from 5 to 2.1 μL in 40 min (see Figure S5), resulting in an evaporation rate of about 1.2×10^−3^ μL⋅s^−1^. For the original foam, the same evaporation rate was assumed and the droplet volume was reduced to 1.7 μL in 20 min, which was assigned to the combined results of evaporation and infiltration. Accordingly, an infiltration rate of about 1.6×10^−3^ μL⋅s^−1^ was obtained for the original foams.

The evaporation effect can be ignored for Type II and III foams, because the lifetime of a droplet is within tens of milliseconds. When a sessile drop is contacted to Type II/III foams, a strong deformation of the droplet was led by the interaction between the collision and the capture actions from the foam, which is presented as a regular pattern. Before being captured, the droplet was spherical, but the solid-liquid interface rapidly expanded in a few milliseconds after contact. As opposed to the Type I foam, the infiltration time of water droplet increased by more than 10,000 times on the Type II/III foams, with the water droplet disappearing within about 30 ms, as shown in Figure 1J.

It is noticed that, in Figure 1J, at the first contact with the Type III foam, part of the water droplet tended to jump into the air due to inertia, but it was pulled down because of the cohesive force, giving rise to the transformation from a ball to a hat shape after 3 ms. The hat-like droplet gradually collapsed and finally disappeared completely. The droplet permeated into the foam under capillary effect, which caused horizontal and vertical imbibitions. As shown in Figure 1K, the horizontal imbibition is conducive to the expansion of the base diameter because of its lubrication effect, whereas the vertical imbibition contributes to the shrinking of the diameter. Therefore, the base diameter then shrunk when the vertical imbibition was dominant. The dynamic evaluation is recorded in Video S1. As only capillary effect was taken into account, the infiltration time of Type III foam is about 4 times greater than that of Type II foam (30 versus 130 ms). The greater rate is caused by better vertical imbibition action in the micro-pores, resulting from the improved hydrophilicity of the TNTs upon crystallization via thermal annealing. More capillary tests can be found in Figures S6 and S7. The average infiltration rate for the micro-droplet is estimated to 173 μL⋅s^−1^ for the Type III foam, which is about 5 orders enhancement when compared with the original foam (173 versus 1.6×10^−3^ μL⋅s^−1^).

Figure 1L shows the static contact angle evolution of water droplet on the Type I, Type IV, and Type V foams. The contact angle decreases exponentially as the exposure time for the original foam (Type I) and the relation between them can be expressed by the fitting formula (Motulsky and Ransnas, 1987):

(Equation 1) *θ* = 120.24–0.0097*t*− 0.059*t*^2^

where *θ* and *t* are the contact angle (degree) and time (minute), respectively. It can be calculated from the formula that the hydrophobic-hydrophilic transition occurred at about 22 min. Also, the rate of reduction of contact angle linearly increases with time, resulting from the increased pinning effect caused by capillary penetration. For the TPFS-treated superhydrophobic foams with amorphous and annealed TNTAs (Types IV and V), contact angle evolution curves are almost identical to each other and can be divided into 3 stages. Their initial contact angles both decrease linearly with time, and these decreasing trends were terminated after around 10 min and then leveled off in the second stage. When the exposure time reached 30 min, the contact angle dropped again quickly. Based on Cassie-Baxter's model (Cassie and Baxter, 1944), the liquid cannot infiltrate into the micro-pores and pits on solid surfaces and the air is trapped under the droplet for superhydrophobic surfaces. The evaporation effect is the single factor affecting the contact angle for the Am- (amorphous) and An- (anatase) TPFS (i.e., Types IV and V) foams. Therefore, as shown in Figure 1L, the initial contact angles were both more than 150° and first deceased to the receding angle *θ*_r_ because of a hysteresis effect that originated from the pinning action on the porous surface. Thereafter, the contact angle remained constant because of the reduced pinning resistance. However, the contact angle dropped again after 30 min, which may be caused by the collapsed air pockets with the liquid permeating into the foams. Therefore, the effective time was 30 min for resistance to imbibition for a micro-droplet. Our results therefore successfully demonstrated the superhydrophilic-to-superhydrophobic transition by a chemical modification with TPFS on the Ti foam with 3D percolative TNTAs.

### 3D Percolative TiO_2_ Nanotube Arrays-Decorated Ti Foam with Millimeter Thickness for Oil-in-Water Emulsion Separation

**Figure 2:**
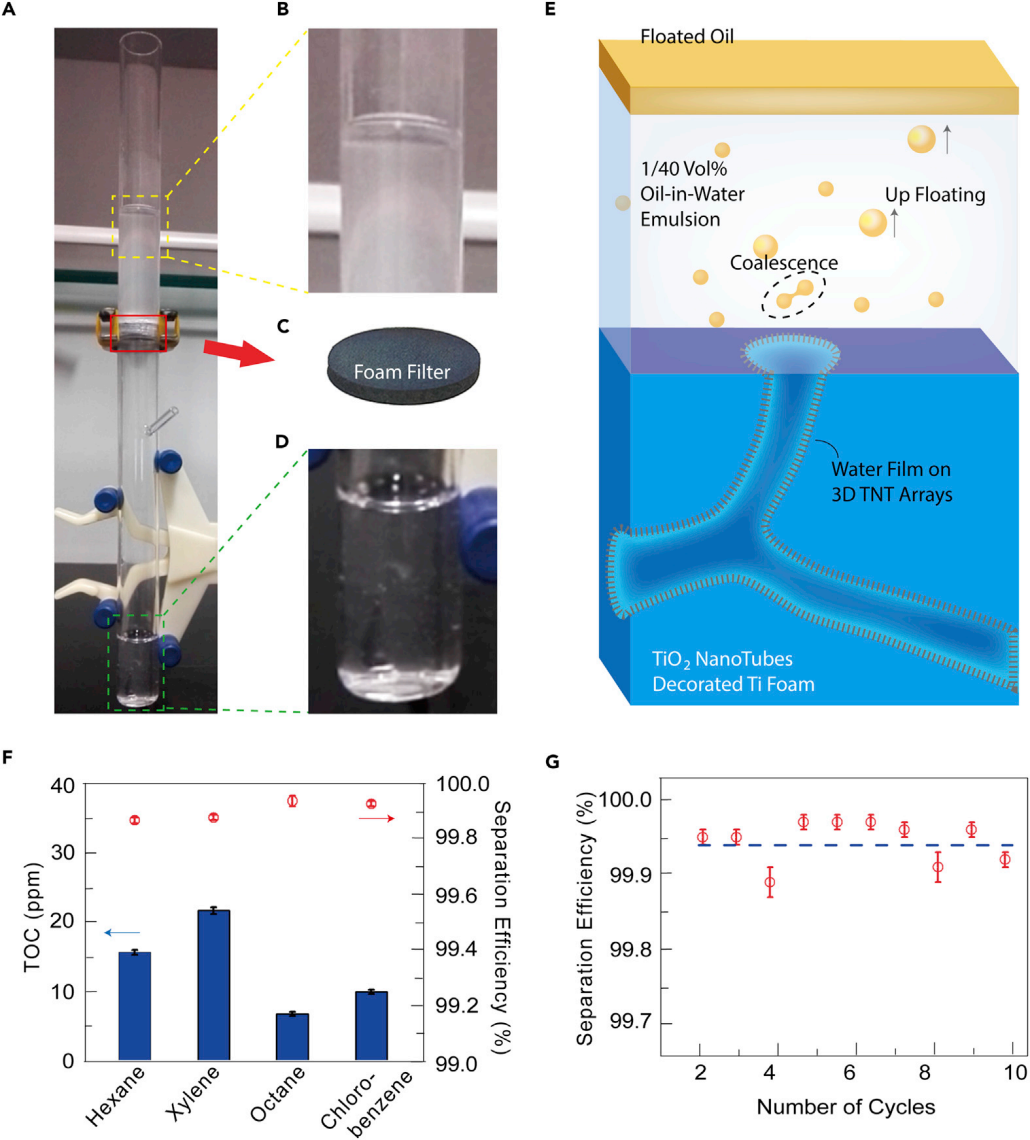
Demonstration of Oil-in-Water Emulsion Separation by 3D-TNTs (A–D) (A) Experimental setup of vertical deployment, where local magnifications are given in (B–D). (E) Illustration showing the separation mechanism. (F) The amount of total organic carbon in filtrates and the corresponding separation efficiency using different emulsions. (G) Recycling tests of the superhydrophilic foam (Type III) employing octane-in-water emulsion. Each of the data point in (F) and (G) was based on at least three samples. See also Figures S6, S8, and S9.

To test the oil/water separation capability of the 3D TNTAs-decorated Ti foams, 4 kinds of oil-in-water emulsions (hexane, xylene, octane, and chlorobenzene) were prepared. A vertical deployment of oil-in-water emulsion separation device is shown in Figure 2A. The upper quartz tube is the feeding container with emulsion, and the lower one is installed to collect the filtrated water, as shown in Figures 2B and 2D. The 3D TNTAs-decorated foam (Type III) with a thickness of 3 mm (Figure 2C) was fixed between 2 tubes for emulsion separation. The separation process is driven by gravity and is recorded in Video S2. A schematic of the separation mechanism of oil-in-water emulsion is illustrated in Figure 2E. Finite oil droplets freely disperse in the emulsion, and their diameter ranges from several microns to tens of microns (Figure S8). When the foam contacts the emulsion, the intrusion pressure of water is decreased (Δp_1_<0), resulting from the superhydrophilic TNTAs, and the foam is completely wetted in the timescale of milliseconds under 3D capillary force (see Figure S9). Those oil droplets moving toward the foam under the downward permeation flow cannot directly pass through the foam because the intrusion pressure of oil is opposite to the flow direction (Δp_2_>0, Figure S9). Therefore, the micron-sized oil droplets gathering near the foam surface tend to coalesce into larger sized droplets (Figure 2E), due to the improved oil droplet density at the surface. The coalesced oil droplets are then released from the surface and float up because of the increased buoyancy. Finally, the droplets float on top of the water and a free oil layer appears, as observed in the process (Figure 2B).

Video S2. Separation Process Driven by Gravity with a Device Shown in Figure 2A, Related to Figure 2 A superhydrophilic Type III foam was used in this video.

To emphasize the importance of the 3D percolative anodization of TNTAs, we compare the oil/water separation performance between the Type III foam and the original Ti foam, as shown in Videos S2 and S3, respectively. In addition, it is essential to also compare with those only anodized at the surface (zone A), while the core of the foam are not coated conformably with TNTs, as seen in many conventional anodized Ti foams (Li et al., 2015c).

Video S3. Separation Process Driven by Gravity with a Device Shown in Figure 2A, Related to Figure 2 An original Ti foam was used in this video.

To demonstrate it more precisely, only one surface in zone A was coated with nanotubes (referred to as single-sided superhydrophilic foam). The visual performance of oil-in-water emulsion separation using the single-sided foam is shown in Video S4, with its water absorption test given in Figure S7 and evolution of water droplet in Figure S10. Type III foam shows the best performance in terms of separation speed and efficiency, wherein the white emulsion turned transparent after separation from the TNTAs-decorated Ti foam (Video S2). For the original and the superficially anodized foams, the filtrate is still turbid with a lot of oil droplets in water, or frequently the foams are even clogged by the emulsion.

Video S4. Visual Performance of Oil-in-Water Emulsion Separation Using the Single-Sided Foam, Related to Figure 2

To quantitatively study the efficiency of oil-in-water emulsion separation, the content of total organic carbon (TOC) in the filtrated water from 4 kinds of emulsions was measured. As shown in Figure 2F, for all emulsions, the TOC content is lower than 25 ppm, and the TOC content is as low as 7.5 ppm for octane-in-water emulsion. The corresponding separation efficiencies are stabilized around 99.9% for different emulsions, and, specially, an efficiency of 99.95% is obtained for octane-in-water emulsion. The recycled separation was carried out for octane-in-water emulsion to evaluate the separation stability of the TNTAs-decorated foam. As shown in Figure 2G the separation efficiency maintained after 10 cycles, which illustrates that the TNTAs-decorated Ti foam has a good cycling stability for emulsion separation.

## Discussion

### Demonstration of a Self-Driven Siphon-like Demulsificator

**Figure 3:**
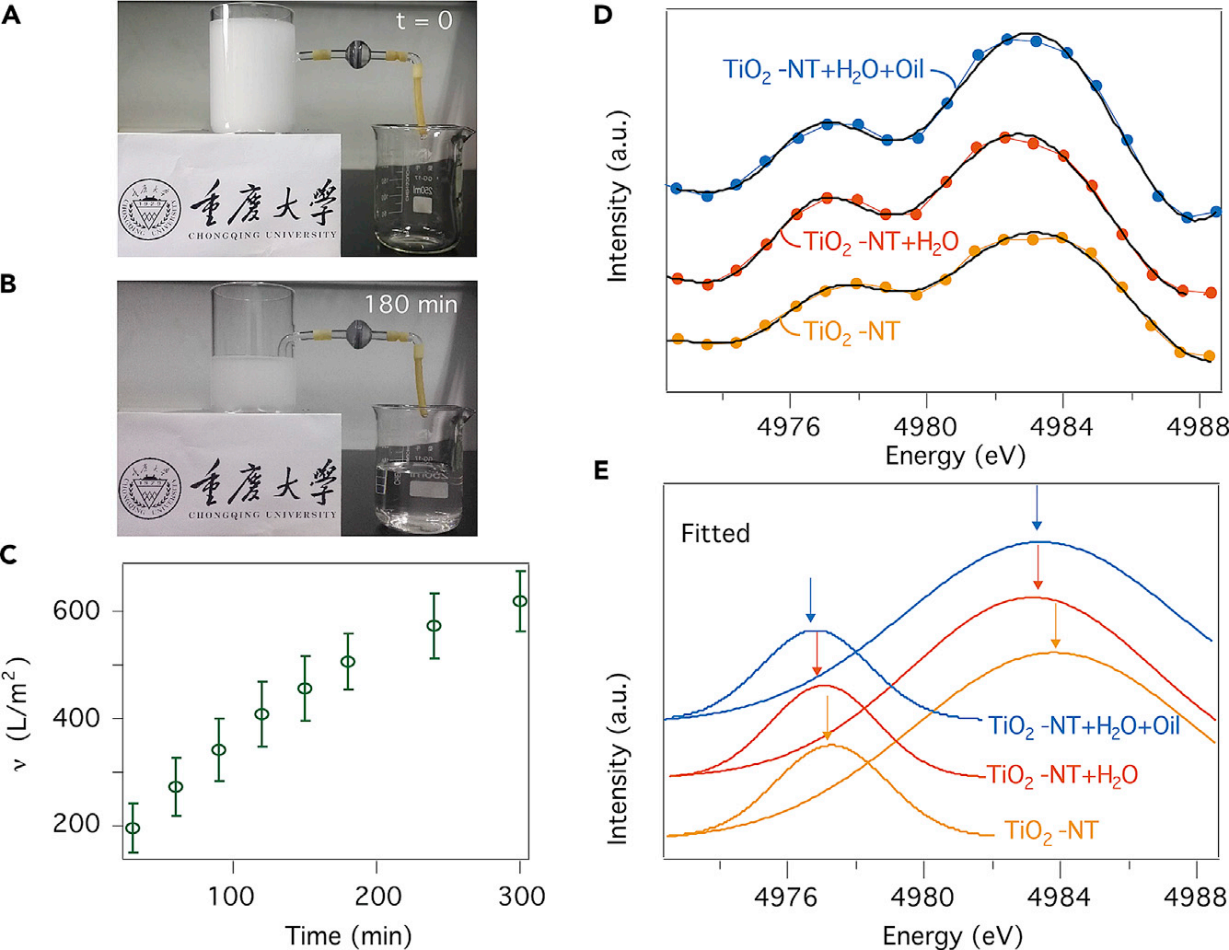
Siphon-like Demulsificator and the XANES Analysis (A and B) An oil filter for octane-in-water emulsion in the configuration of a siphon. (C) Filtrate volume per unit medium area (*ν*) of the prototype siphon filter as a function of time. Each of the data point was based on at least three independent measurements. (D and E) The first derivative of XANES of Ti K-edge for TiO_2_ nanotubes (TiO_2_-NT, yellow) and that immerged in water (TiO_2_-NT + H_2_O, orange) and octane-in-water emulsion (TiO_2_-NT + H_2_O + oil, blue). See also Figures S11–S13.

As a proof of principle for realizing self-driven high-speed demulsificator using the 3-mm 3D TNTAs-decorated Ti foam, we used a simple siphon setup to separate octane-in-water emulsion with droplet size of the order of 10–20 μm. As shown in Figures 3A and 3B and Figure S11 oil-in-water emulsion can indeed be effectively separated by the self-driven Siphon effect (visual tests of the Siphon device as well as capillary forces can be seen in Videos S5, S6, and S7). In our demonstration, a Type III foam having cross section of about 3.1 cm^2^ was used in the link between 2 containers, which leads to a filtration rate of about 120 L⋅m^−2^⋅h^−1^ based on the slope in the linear section (Figure 3C).

Video S5. Visual Performance of Oil-in-Water Emulsion Separation Using the Type III Foam with a Siphon Device, Related to Figure 3

Video S6. Visual Performance of Capillary Force in a Vertical Type III Form, Related to Figure 1

Video S7. Visual Performance of Capillary Force in an Arc-Shaped Type III Form, Related to Figure 1

Furthermore, the compressive stress-strain curves of the original and TNTAs-decorated Ti foams are shown in Figure S12. For the 3D percolative TNTAs-decorated Ti foam, the compressive resistance is in line with that of the original foams. Here, the yield strength of titanium foam reaches 75 MPa, which is much higher than that of other inorganic materials used in the field of oil/water separation (Xu et al., 2012, Biswas et al., 2016). As a comparison, the commonly used organic oil/water separation membrane polyvinylidene fluoride has a very poor compressive property (Cui et al., 2014), whereas the inorganic materials perform relatively better.

It therefore indicates the feasibility of massive demulsification in an industrial scale, namely, by enlarging the area of the foam filter, using the Siphon effect, one can achieve high-speed and high-efficiency oil-in-water emulsions with ultrafine oil droplets (diameter of ∼10 μm), while keeping its mechanical strength and presenting outstanding cycling-stability.

### Characterizations of the Foams Using X-Ray Absorption Near-Edge Spectroscopy

To understand the mechanism of water wettability to the TNTAs on Ti foam, *in situ* X-ray absorption near-edge spectroscopy (XANES) (see Figure S13 for the experimental setup) was used to probe the bonding between TNTs and different liquids. As shown in Figures 3D and 3E, the absorption profile of Ti K-edge (the first derivative of XANES) is illustrated along with Gaussian fits of each peak in the energy window of Ti K-edge. The absorption profile of TNTs shows negligible changes when immerged in octane. This suggests a weak interaction between TiO_2_ and octane, as no functional groups are present in the molecule. In contrast, the profile exhibits substantial red shift upon immerging in water. This is attributed to the bonding between the Ti^4+^ ions at TiO_2_ surface and the OH^−^ groups in water, lowering the chemical states of Ti^4+^ ions (Henderson, 1996, Fujishima and Honda, 1972).

Figure 3E displays a similar red shift once the nanotubes are in contact with the octane-in-water emulsion. It indicates that the water molecules in the emulsion have a high priority of bonding with the nanotubes, in light of their rapid infiltration time (30 versus 150 ms) and high volume ratio (40: 1) when compared with octane. As such, the following mechanism dominates in the process of oil/water separation. The moment the emulsion meets the superhydrophilic foam, a water film is developed immediately in the micro channels via OH^−^ groups. This expels most of the oil droplets at the estuary of the channels, in view of the immiscibility between oil and water. Even though a few droplets of small size are involved, it is difficult for them to pass through the 3D channels of irregular feature, thus being driven out eventually.

### Comparison of the State-of-the-Art Oil/Water Separators

**Figure 4:**
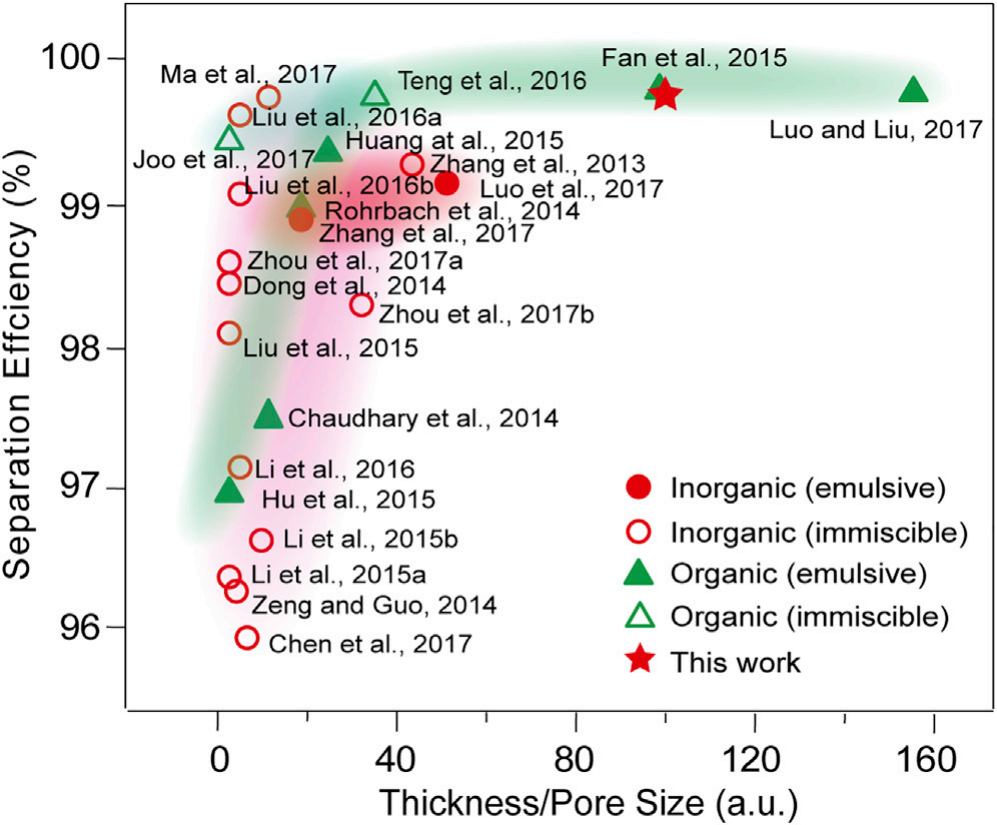
Statistics on Various Demulsificators The diagram compares the oil-in-water emulsion separation properties of the current work with that of other reports. (Chaudhary et al., 2014, Chen et al., 2017, Fan et al., 2015, Hu et al., 2015, Huang et al., 2015, Joo et al., 2017, Li et al., 2015a, Li et al., 2015b, Li et al., 2016, Liu et al., 2016a, Liu et al., 2016b, Liu et al., 2015, Luo et al., 2017, Ma et al., 2017, Rohrbach et al., 2014, Teng et al., 2016, Zeng and Guo, 2014, Zhang et al., 2013, Zhang et al., 2017, Zhou et al., 2017b).

To further illustrate the separation performance, the emulsion separation efficiency of the 3D percolative TNTAs-decorated Ti foam was compared with that in other studies, as shown in Figure 4. The abscissa is a dimensionless value, which is the ratio of the thickness to the average pore size for porous separation materials. This ratio, however, represents highly the essence of the desired filter, as for oil-in-water emulsion separation, the increase in the thickness is usually beneficial to enhance the separation efficiency due to the longer micro channel, whereas the smaller pore size is expected because the screening effect is stronger for oil droplet.

A reasonable combination of the thickness and pore size is required, because the measures can reduce the flow and channel clogging problems may occur. The result shows that the 3D percolative TNTAs-decorated Ti foam have an outstanding oil/water separation performance whether in organic or inorganic oil/water separation material system. Especially, the separation efficiency is also dazzling among the materials for oil-in-water emulsion separation.

### Conclusion

We have demonstrated a 3D percolative anodization technique with which high-porosity titanium framework with thickness reaching a few millimeters can be conformably decorated with superhydrophilic TNT vertical arrays. These 3D percolative superhydrophilic micro channels can serve as a “highway” for water but prevent the transport of oil molecules. This results in a reusable oil/water separator that filters oil droplets of average diameter 10 μm, with >99.95% efficiency and strong mechanical durability. Moreover, those 3D percolative micro channels can be further turned into superhydrophobic by TPFS treatment, which may expand the application of TNTAs-decorated Ti foam into another limit of the water-in-oil emulsion. Our studies show that nanostructure-modified percolative metal frameworks hold great promise for future demulsification technology.

## Methods

All methods can be found in the accompanying Transparent Methods supplemental file.

## Supplemental Information

Document S1. Transparent Methods, Figures S1–S13, and Table S1
