# Supplementary material for: Nanostructured Three-Dimensional Percolative Channels for Separation of Oil-in-Water Emulsions
Source: iScience. 2018 Aug 13;6:289–98. doi: 10.1016/j.isci.2018.08.004 (PMC6137911; doi:10.1016/j.isci.2018.08.004)
Supplement: Document S1. Transparent Methods, Figures S1–S13, and Table S1 [file mmc1.pdf]

ISCI, Volume *6*

Supplemental Information

Nanostructured Three-Dimensional Percolative Channels for Separation of Oil-in-Water Emulsions

Jian Jin, Xiaoli Zhao, Yong-Hua Du, Mei Ding, Chengjie Xiang, Ning Yan, Chuankun Jia, Zheng Han, and Lidong Sun

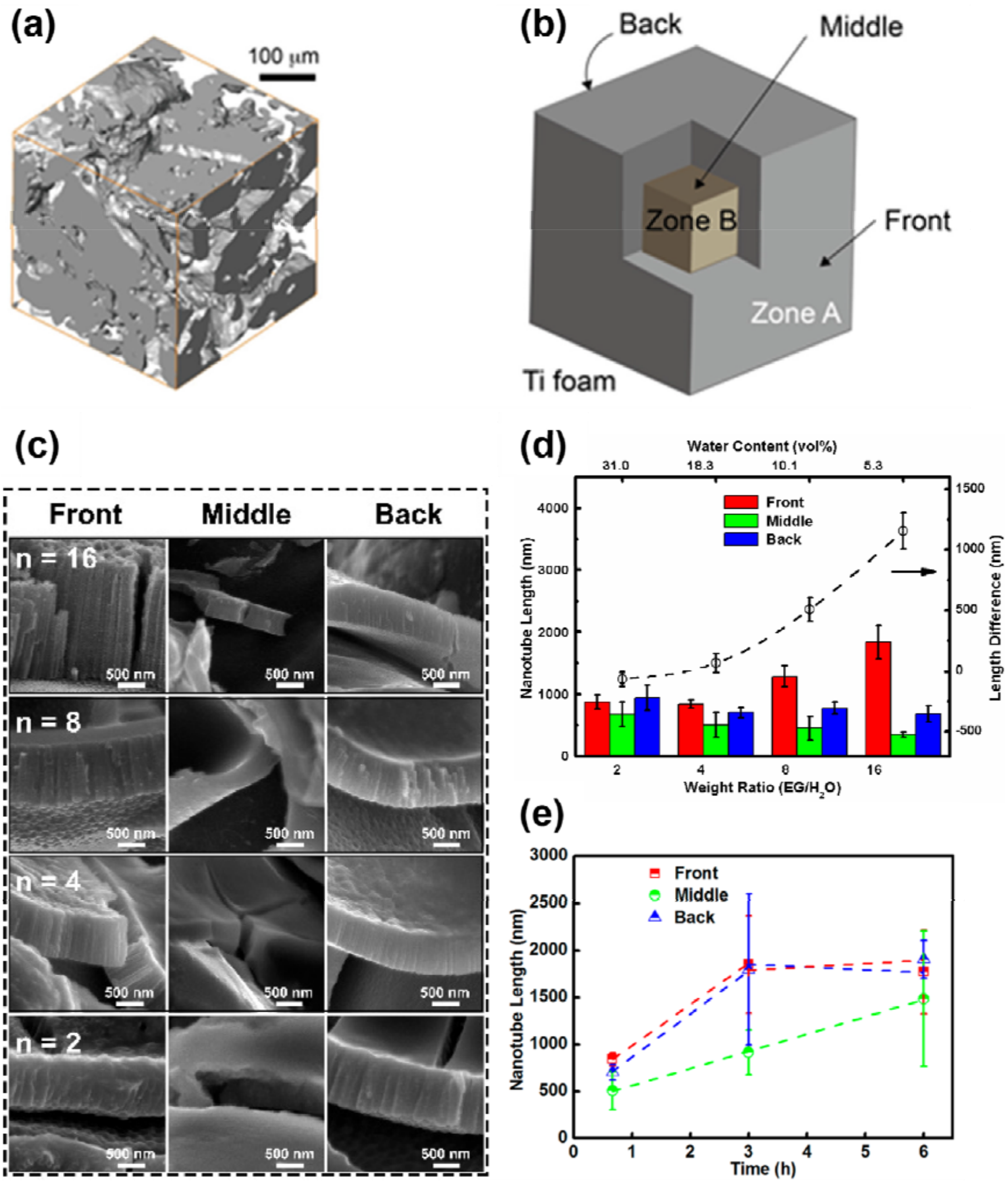

Figure S1. Structure optimization of TNTAs on Ti foams, related to Figure 1. (a) Micro-CT image of the Ti foam; (b) Schematic of outer and inner regimes, denoted as Zone A and Zone B, as the inner Zone B is more difficult to be fully covered with TiO_2 nanotubes during the anodization process; (c) SEM images of the three representative regions (Front: the regions close to the Ti foam surface that faces the cathode, Back: the regions close to the other surface, Middle: the regions in between, as illustrated in (b)) in Ti foams of 3 mm in thickness, which were obtained using electrolytes of different weight ratios (n=2, 4, 8, 16) of ethylene glycol (EG) to water; (d) Corresponding nanotube length (left) and length difference between the Front and Back (right) under the conditions in (c); (e) Time dependence of tube length at different positions when n=4 for different durations. Each of the tube length is based on 3−5 samples and at least 10 FESEM images for each sample.

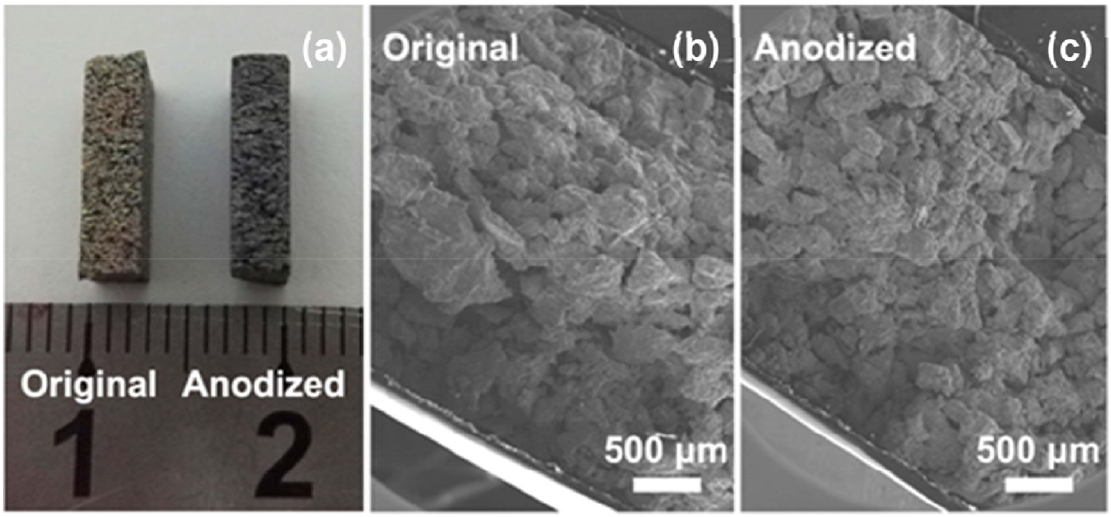

Figure S2. Morphologies of original and anodized Ti foams, related to Figure 1. (a) Comparison of optical morphology of original and anodized Ti foam used in this work. Their low-magnification SEM images are given in (b) and (c), respectively.

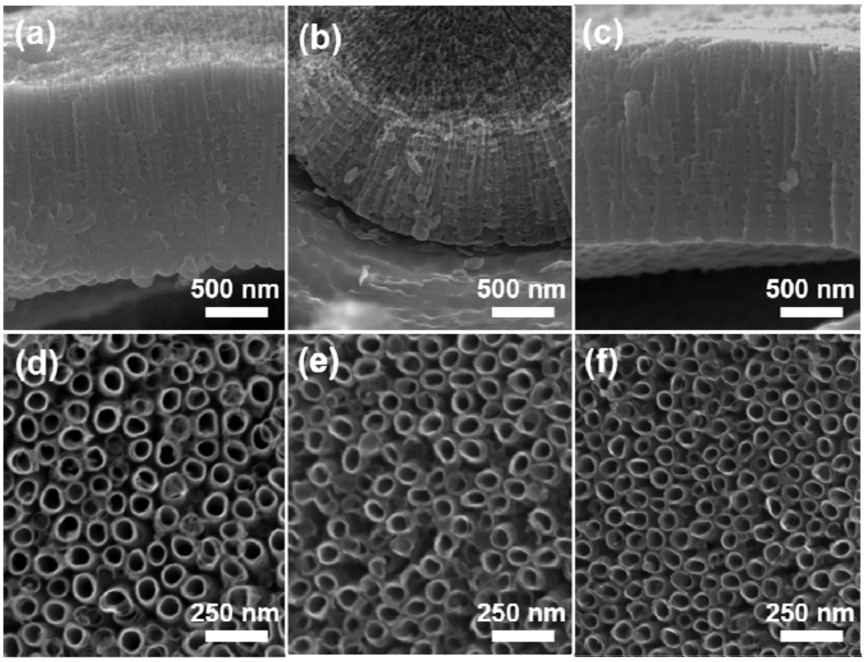

Figure S3. Features of TNTAs used for oil-water separation, related to Figure 1. Cross-sectional (a–c) and surface (d–f) FESEM images of the TiO_2 nanotube arrays prepared in ethylene glycol containing 18.3 vol% water (weight ratio n=4) and 0.3 wt% NH_4F at 40 V and 22 °C for 3 h, with the distance between anode and cathode being kept at 12 mm. Such conditions were employed to fabricate conformal nanotube coatings inside Ti foams for subsequent oil-water separation application. The panels from left to right are nanotube coatings at the front (a, d), middle (b, e), and back (c, f), respectively.

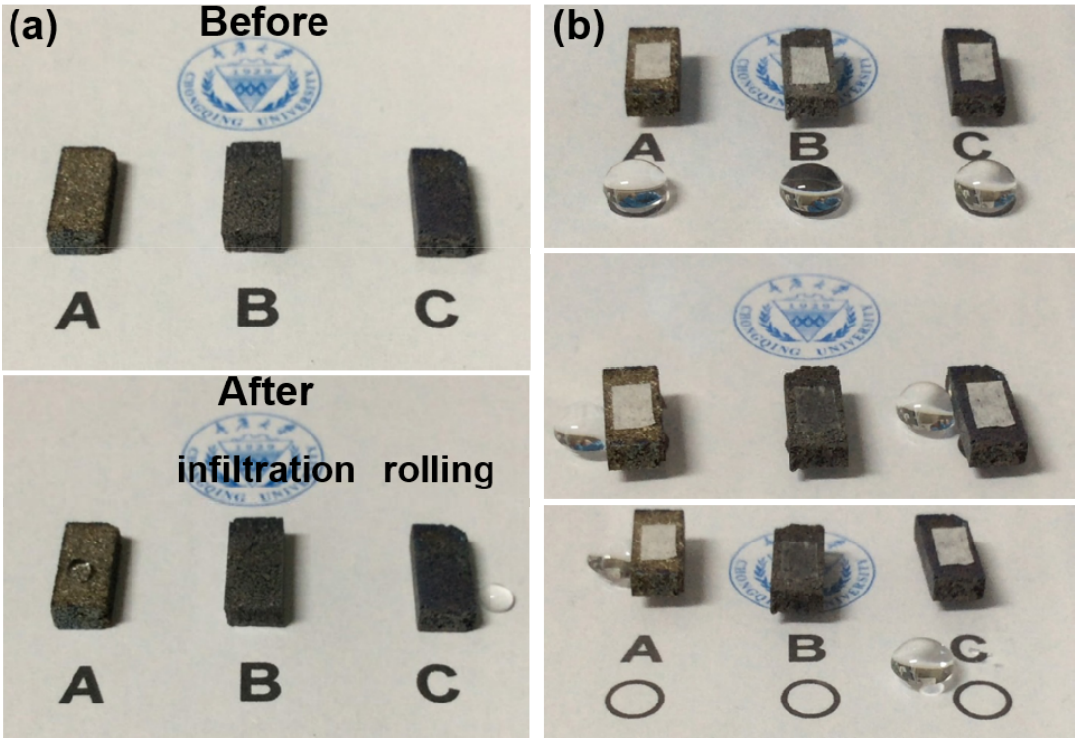

Figure S4. Surface wettability characteristics of different Ti foams, related to Figure 1. Demonstration of top-down infiltration (a) and bottom-up absorption (b) of water droplets for different Ti foams: A, original; B, superhydrophilic; C, superhydrophobic. A piece of filter paper was put on top of each foam in (b).

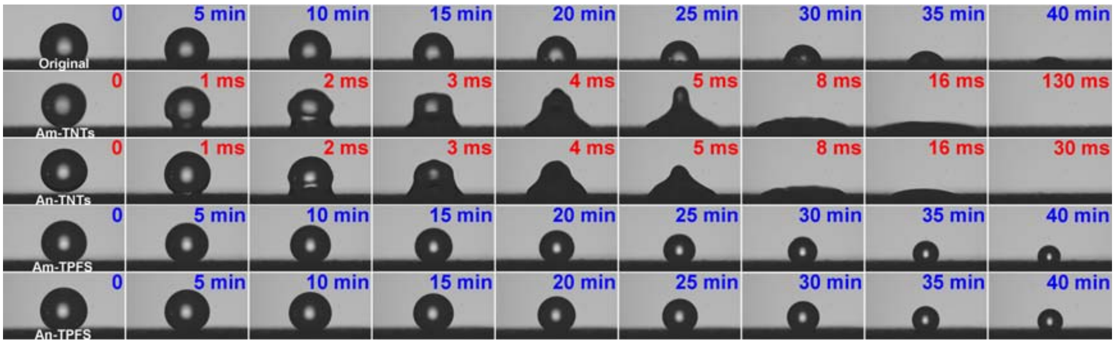

Figure S5. Water droplet evolution on different Ti foams, related to Figure 1. Time sequence of movie frames showing the evolution of water droplet on different foams in Table S1: Type-I (original, 1st row), Type-II (Am-TNTs, 2nd row), Type-III (An-TNTs, 3rd row), Type-IV (Am-TPFS, 4th row) and Type-V (An-TPFS, 5th row).

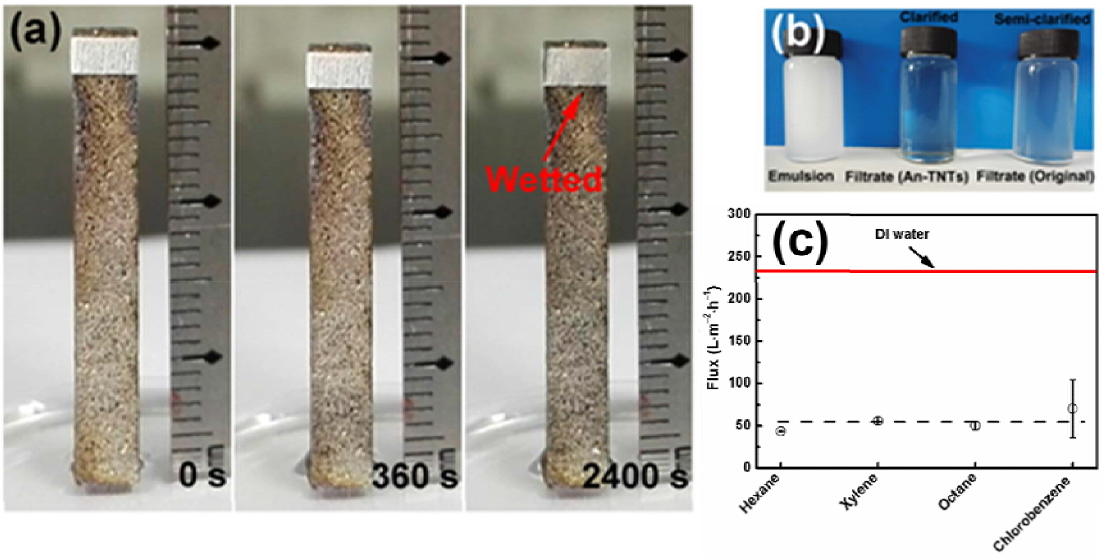

Figure S6. Water absorption effect of original Ti foam and oil-in-water emulsion separation performance of superhydrophilic foam, related to Figures 1 and 2. (a) Demonstration of bottom-up water absorption (capillary effect) for an original titanium foam of about 30 mm in height; the foam was in contact with water at the bottom and wrapped with filter paper at the top to detect the wetting height and speed by water; (b) Photographs of original octane-in-water emulsion (volume ratio 1:40) (left) and the filtrate obtained using superhydropilic (middle) and original (right) foams; (c) Infiltration capability of water and different emulsions using the setup in Figure 2 (see main text) with superhydrophilic foams.

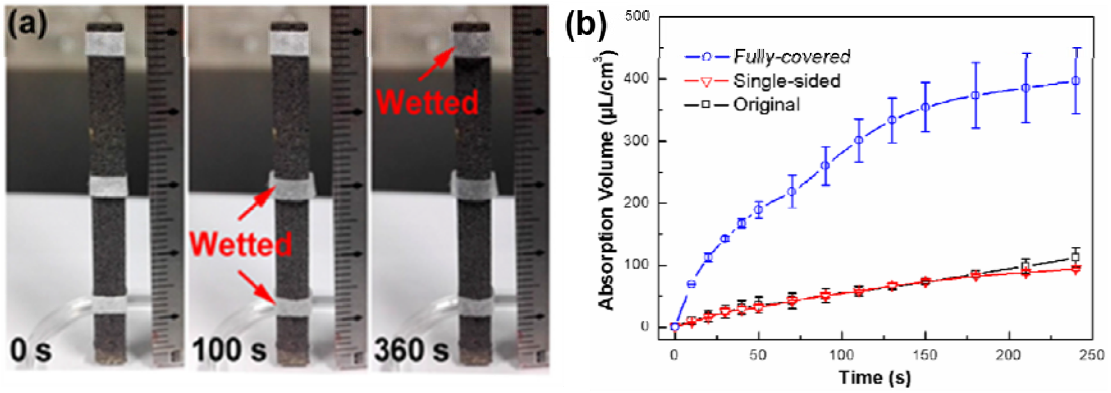

Figure S7. Water absorption property for different foams, related to Figure 1. (a) Demonstration of bottom-up water absorption for a superhydrophilic foam of about 50 mm in height; (b) the relation of absorption volume with time for the original foam (Original), the foam fully covered with nanotubes (Fully-covered), and the foam with only one surface layer being covered with nanotubes (Single-sided). See also Movie 4-5. For the Fully-covered foam (Type-II and Type-III), the water was instantaneously absorbed into the titanium foam and infiltrated to the upper surface under the capillary force, resulting in the wetting of the paper. As for the Original (Type-I) and Single-sided foams, the absorption process was extremely slow.

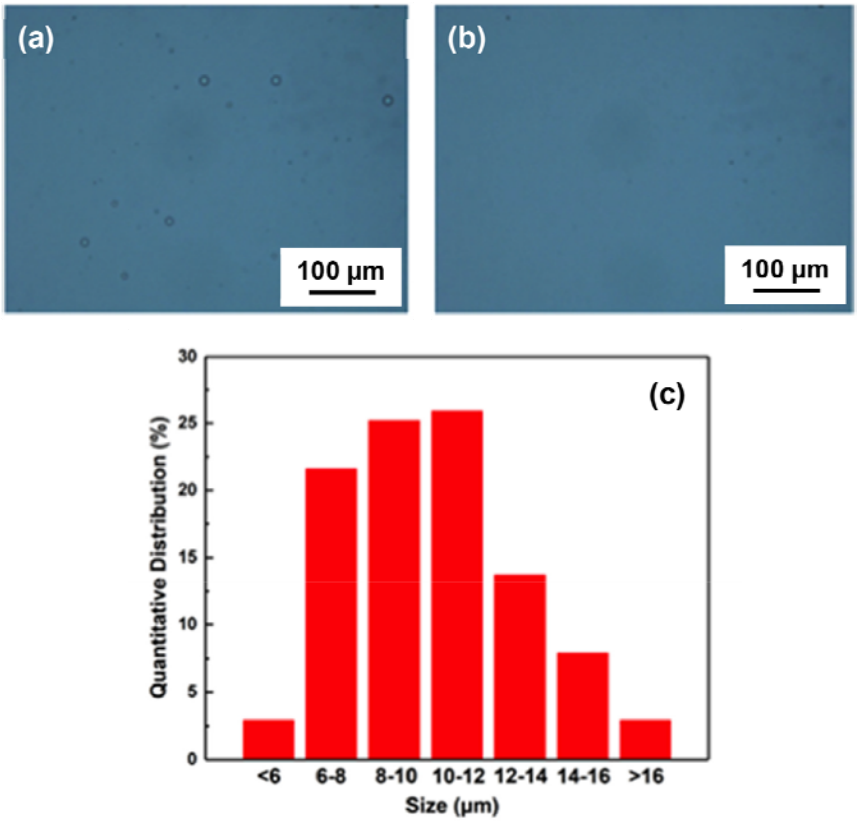

Figure S8. Oil droplet size in octane-in-water emulsion, related to Figure 2. Optical images of octane-in-water emulsions before (a) and after (b) filtration by superhydrophilic foams (Type-III). Corresponding statistics of the droplet diameter is given in (c).

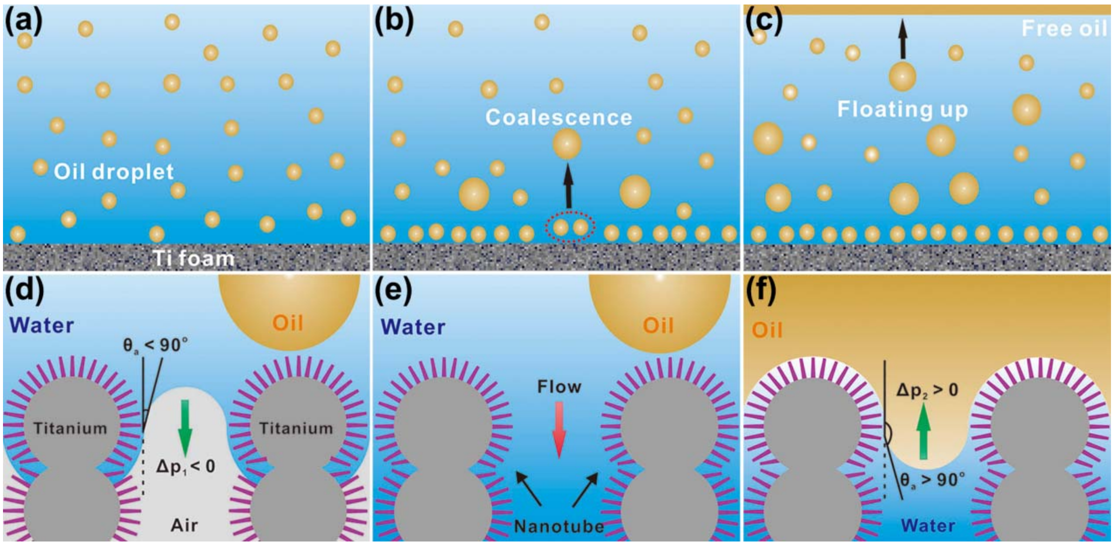

Figure S9. Demulsion mechanism, related to Figure 2. Illustration of the demulsion process (a-c) and the corresponding micromechanism (d-f) using superhydrophilic foams (Type-III).

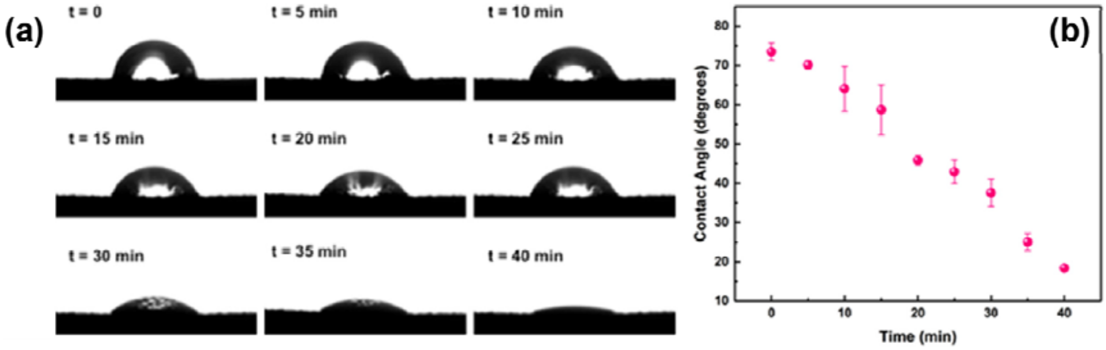

Figure S10. Evolution of water droplet on the single-sided foam, related to Figure 1. (a) Time sequence of movie frames showing the evolution of water droplet on the foam, with only one surface layer being covered with nanotubes (Single-sided); (b) Contact angle of the water droplet as a function of time for the foam tested in (a).

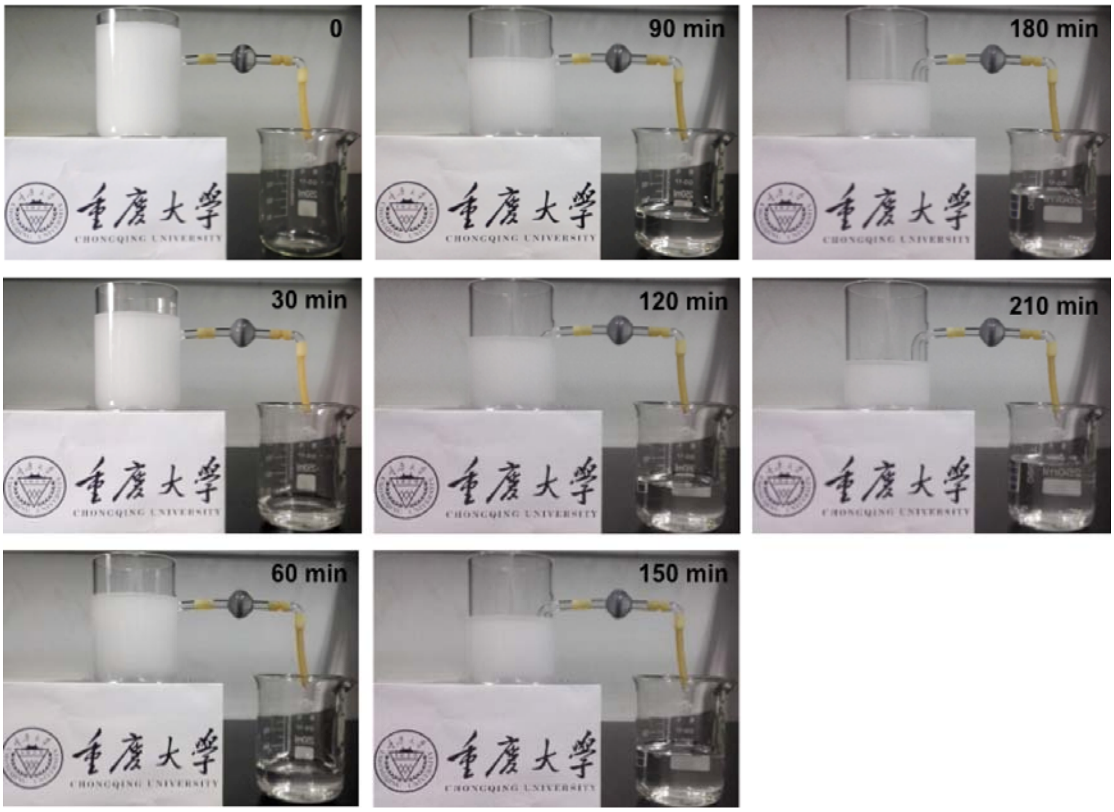

Figure S11. Siphon-like demulsificator for oil/water separation, related to Figure 3. Siphon test using the superhydrophilic foam (Type-III) coated with TiO_2 nanotube arrays in the 3D percolative channels, related to Fig. 3.

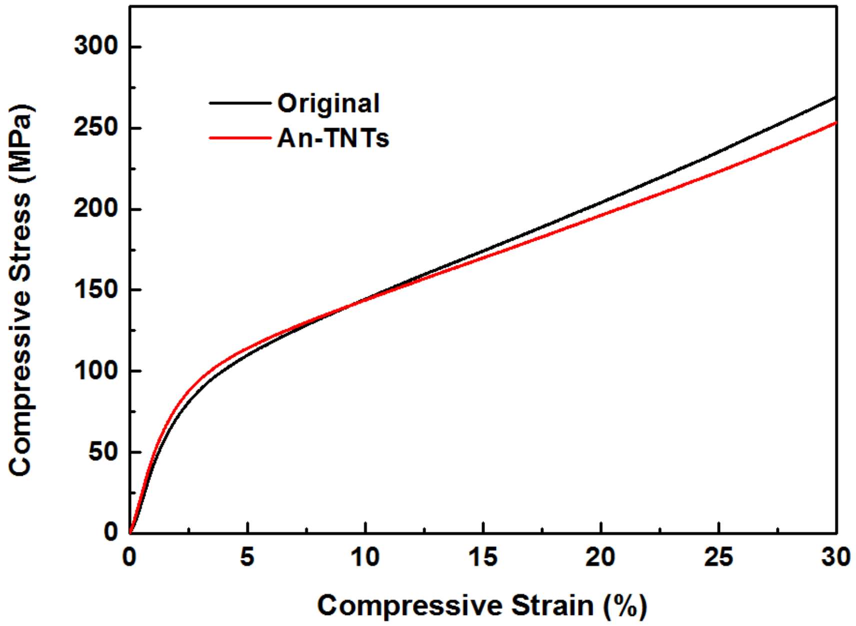

Figure S12. Mechanical properties of different foams, related to Figure 3. It is seen that the An-TNTs foam keeps almost the same mechanical properties as the original Ti foam, indicating that our demulsificator is of strong durability with respect to many such as organic oil-water separators.

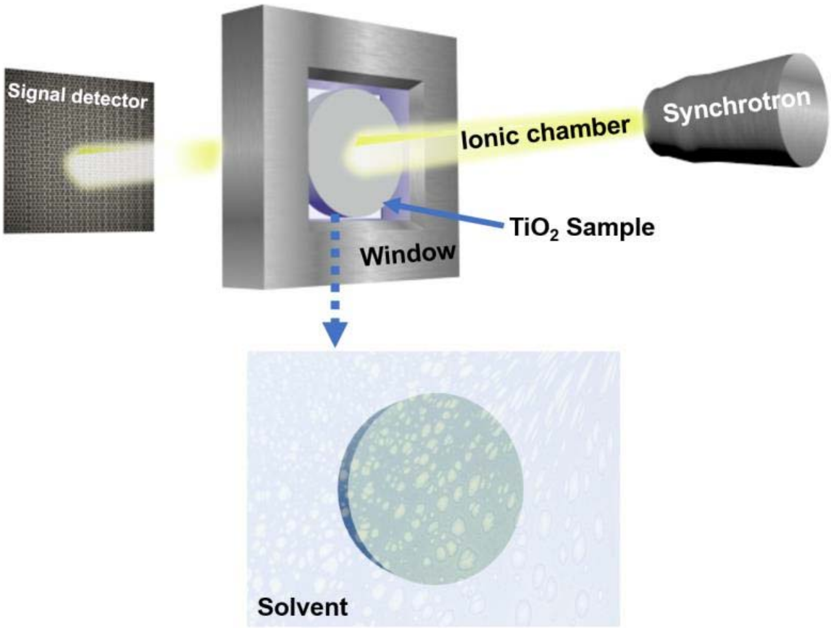

Figure S13. Experimental setup for in-situ XANES measurement, related to Figure 3. The samples were immersed in liquids during the measurement using a customized holder.

Table S1. Summarization of different Ti foams studied in this work, related to Figure 1

	Foam description	Wettability
Type-I	Original Ti foam	Hydrophobic
Type-II	Ti foam decorated with amorphous TNTAs	Superhydrophilic
Type-III	Ti foam decorated with anatase TNTAs	Superhydrophilic
Type-IV	Ti foam decorated with amorphous TNTAs and treated by TPFS	Superhydrophobic
Type-V	Ti foam decorated with anatase TNTAs and treated by TPFS	Superhydrophobic

Transparent Methods

Fabrication of 3D percolative superhydrophilic channels. Titanium foams (thickness: 3 mm, porosity: 38 vol%, accuracy: 30 μm) were ultrasonically cleaned in isopropanol, alcohol, sulfuric acid and deionized water for 15 min each, and finally dried in a nitrogen steam. The TiO_2 nanotube arrays were produced by anodizationin ethylene glycol (EG, anhydrous, 99.8%, Sigma-Aldrich) containing deionized water ($EG_{(wt)}/H_2O_{(wt)} = n$, n = 2, 4, 8, 16) and 0.3 wt% ammonium fluoride (NH_4F, 98%, ACS reagent, Sigma-Aldrich) under a constant voltage of 40 V (Kethley 2450 SourceMeter as the power supply) at room temperature (~22°C) for 40 min. A longer anodization durationwas employed in the electrolyte solution with n=4 to achieve open-ended TNTs. The resulting titanium foams were then annealed at 450°C for 3 h in furnace (LE140K1BN, Nabertherm) for crystallization. For superhydrophobic treatment, the foams were immersed in *n*-hexane (99%, Adamas) containing 0.5 vol% trichloro (1*H*, 1*H*, 2*H*, 2*H*-perfluorooctyl) silane (TPFS, 97%, Sigma-Aldrich) for 30 min, and then annealed in nitrogen atmosphere at 110°C for 1 h.

Characterizations of the TNT behaviors. The surface and cross-sectional morphologies of TNTs were examined by field-emission scanning electron microscope (FESEM, FEI Nova 400). The length of the nanotubes was directly determined by the ImageJ software. The evolutions of the sessile droplets on the foams were recorded by a high speed video camera. The initial volume of the droplet was about 5 μL. In absorption test, the foam (about 50 mm in height) fully covered with TNTs was in contact with water at the bottom. The absorption volume V_t ($\mu L \cdot cm^{-3}$) was determined by:

$$V_t = (w_t - w_0) / \rho V_0$$

where w_0 and V_0 are the weight and volume of the foam, w_t is the total weight of the foam after absorption for t min, and ρ is the water density.

Oil-in-water emulsion separation related methods. The resulting superhydrophilic foams were fixed between two quartz glass tubes. Four kinds of oils, including hexane (95%, anhydrous, Sigma-Aldrich), xylene (99%, anhydrous, Sigma-Alidrich), octane (99%, anhydrous, Sigma-Alidrich) and chlorobenzene (99.8%, anhydrous, Sigma-Alidrich), were applied for oil-in-water emulsion separation. All emulsions were prepared by ultrasonically mixing oil with water (volume ratio of 1/40) for 30 min. The emulsions were then poured into the upper tube, and the separation was only driven by gravity. After separation, the foam was washed with ethanol and dried in a vacuum oven at 50°C, and reused for recycling test. The filtrate water was collected for total organic carbon test (TOC, SSM-5000A, Shimazu). The separation efficiency was computed by oil rejection coefficient R, as follows (Xue et al., 2011):

$$R = [1 - (C_p / C_o)] \times 100\%$$

where C_o and C_p are the oil concentration of the original oil/water mixture and the collected water after one time separation, respectively.

Supplemental References

Xue, Z., Wang, S., Lin, L., Chen, L., Liu, M., Feng, L., and Jiang, L. (2011). A novel superhydrophilic and underwater superoleophobic hydrogel-coated mesh for oil/water separation. Adv. Mater. *23*, 4270-4273.
